# Relational Memory: A Daytime Nap Facilitates the Abstraction of General Concepts

**DOI:** 10.1371/journal.pone.0027139

**Published:** 2011-11-16

**Authors:** Hiuyan Lau, Sara E. Alger, William Fishbein

**Affiliations:** 1 Department of Psychology, The City College of the City University of New York, New York, New York, United States of America; 2 Center of Excellence on Brain Aging, Langone Medical Center, New York University, New York, New York, United States of America; Université Pierre et Marie Curie, France

## Abstract

It is increasingly evident that sleep strengthens memory. However, it is not clear whether sleep promotes relational memory, resultant of the integration of disparate memory traces into memory networks linked by commonalities. The present study investigates the effect of a daytime nap, immediately after learning or after a delay, on a relational memory task that requires abstraction of general concept from separately learned items. Specifically, participants learned English meanings of Chinese characters with overlapping semantic components called radicals. They were later tested on new characters sharing the same radicals and on explicitly stating the general concepts represented by the radicals. Regardless of whether the nap occurred immediately after learning or after a delay, the nap participants performed better on both tasks. The results suggest that sleep – even as brief as a nap – facilitates the reorganization of discrete memory traces into flexible relational memory networks.

## Introduction

Accumulating evidence suggests that sleep, even as brief as a daytime nap, is important for processing different forms of memory [1]–[3], including relational memory [4]–[7]. Relational memory arises from discretely acquired memory traces, integrated into a network through commonalities, thus allowing flexible applications of past experience to a novel but relevant problem [8]. This flexibility of relational memory parallels an important aspect of learning – generalization. Given that the abstraction of generality also critically depends on the integration of fragmentary information, it may be a product of relational memory processing. Hence, one focal point of the present study is whether the benefits of daytime napping on relational memory extend to the abstraction of general concepts.

Few studies have examined the effect of sleep on generalization. One study showed that, after sleep, the extinction of fearful memory was generalized to unextinguished conditioned stimuli [9]. A second correlational study found that infants who napped were better able to generalize an abstract rule in an artificial language [10]. However, the evidence was limited and a causal link between napping and generalization on a higher cognitive level was lacking.

The present study represents the first experimental investigation of the relationship between daytime napping and the abstraction of general concepts. Participants learned English meanings of Chinese characters that shared ideographic components called radicals, each of which represented a general semantic concept. Characters sharing the same radical had related meanings. In order to assess generalization, participants were later asked to match unstudied characters, with previously seen radicals, to their English meanings and to explicitly state the concepts represented by the radicals. Participants who napped were hypothesized to have better relational memory, namely superior performance in both tasks.

Another objective of the study is the temporal separation between learning and the start of the post-learning nap. The nap window began either immediately after learning or after a delay, the duration of which approximated that of the nap period. At the same time, each nap group was accompanied by a wake control group. This manipulation was performed for two reasons: First, it permits examination of a long-standing debate about whether the beneficial effect of sleep on memory is based on active mechanisms or simply a reduction of external sensory stimulation [11], [12]. If the latter were true, the delayed nap group, with the same amount of wakefulness as the immediate wake group, would show no benefit from napping. Alternatively, both the immediate and delayed nap groups would show enhanced relational memory.

The delay allows for another important investigation – how soon after learning must sleep occur in order to benefit relational memory? On one hand, sleep may simply be a state that optimizes eventual memory processes following acquisition. In this case, the effect of sleep would be the greatest immediately following learning and decay with increasing delay. On the other hand, there may be active mechanisms during sleep, aiding memory processing regardless of the time of sleep onset. The majority of previous research used paradigms in which sleep occurred immediately after learning. Only recently has the possible effect of delayed sleep been suggested [13], [14]. Using both immediate and delayed groups, our experiment examined whether the relationship between daytime napping and relational memory is dependent on their temporal proximity.

## Methods

### Participants

Fifty-eight undergraduates were originally recruited from the City College of New York. They were in good health, had normal or corrected to normal vision, and free of medications that might influence sleep. Each participant kept a sleep log for a week prior to the study in order to exclude those with an irregular sleep schedule. Furthermore, non-native English speakers and those with previous experience with ideographic languages such as Chinese characters and Japanese Kanji were excluded due to the nature of the tasks. Participants were asked to refrain from caffeine and alcohol during the 24 hours preceding participation. Out of the original fifty-eight participants, seven were excluded from data analysis for the following reasons: failure to comply with instructions (1 immediate wake participant), failure to fall asleep or excessively fragmented sleep (1 immediate nap and 3 delayed nap participants), falling asleep during the delay (1 delayed nap participant), and admitting to having previous knowledge of Japanese Kanji after completing the tasks (1 delayed nap participant). The remaining fifty-one participants (35 females) were on average 18.98 years of age (range: 18–28). There were four experimental groups: immediate nap (n = 15, 11 females)), immediate wake (n = 14, 9 females), delayed nap (n = 11, 7 females), and delayed wake (n = 11, 8 females). All participants signed a written informed consent. The study was approved by the City College of New York IRB.

### Procedure and stimuli

**Figure 1:**
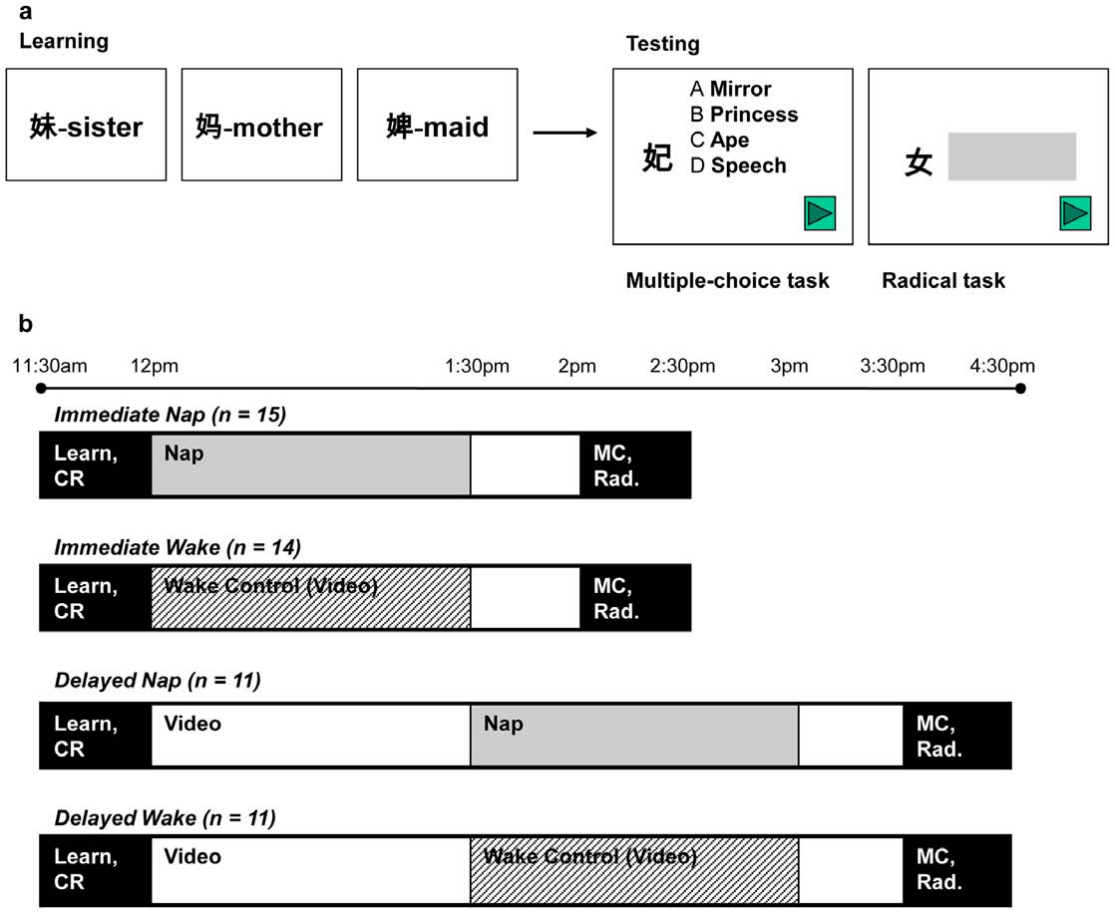
Schematic description of tasks and experimental design. *(a)* Sample stimuli and items from the tasks. *Left:* Individual characters, sharing the same radical, presented in the learning phase. *Right:* A sample item in the multiple-choice task and an isolated radical in the radical task. *(b)* A summary of experimental design and protocol. *CR* – cued recall task; *MC* – multiple-choice task; *Rad.* – radical task.

Participants learned English meanings of twenty-one Chinese ideographs called characters, organized into seven conceptual groups. There are three characters that shared a common conceptual radical in each group (*figure 1a*), and therefore they had related meanings. The characters were presented in a pseudo-random order such that characters with the same radical were not closely positioned. Following two run-through exposures to the characters, participants were given a cued recall task in which they attempted to type the English meaning of a given Chinese character. There were a total of fourteen characters in the cued recall task. The entire learning phase took about 30 minutes to complete.

After learning, each participant stayed in a sleep chamber. Nap participants were permitted an approximately 90-minute opportunity within the retention period to attempt napping. If participants obtained SWS or REM sleep they were allowed to sleep until the SWS or REM sleep period was coming to an end. Participants were awakened from sleep stages 1 or 2 when possible. Therefore the 90-minute window was slightly expanded for some participants to accommodate the procedure of waking up participants.

Wake participants, during the entire retention period, and delayed nap participants, during the pre-nap delay, watched videos about ocean life in the sleep chamber.

All participants were monitored online when they were in the sleep chambers by a digital EEG acquisition software (Gamma System-Grass/Telefactor™) using a five-channel polysomnographic montage, which included electroencephalography, electro-oculography, and chin-electromyography channels. The sleep recordings were scored using the international criteria of [15].

Approximately half an hour after nap participants were awakened, all participants matched English meanings to given characters in a multiple-choice task (*figure 1a*). Importantly, this task consisted of a mixture of seven old characters and twenty-one new characters, which the participants had not studied but consisted of the seven previously seen radicals. There were four answer choices for each given character, one of which was the correct English meaning and the other three distracters. The choices were selected so that if the participants had abstracted the general concept of the radical, they would be able to deduce the correct answer even if they had not studied the given character. Therefore, there were two components in this task – direct associative recognition memory of the old characters and expression of relational memory in the new characters.

Lastly, participants were shown the seven radicals in isolation and were asked to type their meanings (radical task, *figure 1a*). For a given radical, participants were given three points if they typed the correct meaning, two points if the correct meaning of this radical was among the answers but mismatched to a different radical, one point if they typed the meaning of a specific character that contains the radical instead of the general concept of the radical itself. This task aimed to assess the abstraction of a general concept. Therefore, scores for different types of responses were ranked to reflect this priority.

All stimuli and tasks were presented on a 50 cm-monitor in the format of Microsoft PowerPoint slides. All words were presented in 40-point font. Participants responded using the keyboard or mouse. In all tasks, participants were instructed to guess even if they were unsure of the answer.

For a summary of the protocol and design, see *figure 1b*.

Additional to the tasks, each participant completed a demographic questionnaire, an Epworth Sleepiness Scale [16], and the vocabulary subset of the Multidimensional Aptitude Battery-II (MAB-II) [17] at the beginning of the study. The Epworth Sleepiness Scale is a measure of general daytime sleepiness that assesses participants' tendency to fall asleep in various situations. The MAB-II is a standardized paper-and-pencil test commonly used to assess intelligence. Only the vocabulary subset was used in the present study to assess participants' verbal aptitude, which might be a factor in ability to encode the English meaning of the Chinese characters. It also has the highest correlations with intelligence measured by the Wechsler Adult Intelligence Scale. The subtest took 7 minutes to complete.

At the end of the study, participants were asked to rate their motivation in performing the tasks on five-point Likert scales. The average rating was calculated for analyses. They were also asked to rate the difficulty of each task and their interest in the entire study on a scale from 1 to 10, one being the minimal level and ten being the maximal level.

## Results

### Epworth Sleepiness Scale Scores

Data from two participants, one from the immediate nap group and one from the delayed nap group were missing. Analysis on the available data revealed no difference in general daytime sleepiness between the four groups (One-way ANOVA, F_3,45_ = 0.88, p = 0.46).

### MAB-II Vocabulary Subset Scores

Intelligence scores measured by the MAB-II vocabulary subtest were standardized and Z-scores were calculated and used in the subsequent analysis. There was no difference in this intelligence measure between the four groups (One-way ANOVA, F_3,47_ = 1.35, p = 0.27).

### Post-Study Report of Motivation, Task Difficulty, and Interest in the Study

Between-group comparisons showed that the four groups were comparable in each subjective measure (One-way ANOVAs, all p values >0.35).

### Sleep Data

The delayed nap s had significantly longer total sleep time (77.45±3.81 minutes, mean ± SEM) compared to the immediate nap participants (63.17±2.93 minutes). This may be due to greater tendency of the delayed nap participants to enter SWS and REM than the immediate nap participants (72% of the delayed nap participants compared to 53% of the immediate nap participants) although the difference did not reach statistical significance (Chi-square, p = 0.18). Hence, a bigger proportion of the delayed nap participants were allowed to nap longer and to cycle out of SWS or REM before they were woken up. No difference was found in any other sleep parameter.

For a summary of sleep parameters across groups, see *table 1*.

**Table 1:** Summary of sleep parameters (mean ± SEM) across the two nap groups (immediate vs. delayed).

	Immediate Nap (n = 15)	Delayed Nap (n = 11)
Sleep onset	8.63±1.18	6.82±2.21
Total sleep time *	63.17±2.93	77.45±3.80
Sleep efficiency (%, = total sleep time/total time in bed)	79.79±2.89	80.82±3.50
Stage 1	5.93±1.31	6.27±0.92
Stage 2	31.33±2.59	36.09±4.33
Slow wave sleep	19.77±4.11	22.18±4.03
REM sleep	5.67±1.93	12.86±3.85

All means and SEMs are reported in minutes except sleep efficiency. Asterisk represents significant group difference (* p<0.05).

No significant correlation between sleep parameters and task performance was detected.

#### Cued Recall Task

Performance on the cued recalled task at learning was measured by the number of correct English meanings recalled. Average performance ± SEM for each group was, respectively, as followed: immediate nap = 8.53±0.85, immediate wake = 8.36±0.75, delayed nap = 8.00±1.00, delayed wake = 8.26±0.41. There was no group difference in recalling English meanings of individual characters in the learning phase (One-way ANOVA, F_3,47_ = 0.09, p = 0.96).

Performance on the cued recall task was used as a covariate in the following analyses of post-nap tasks to control for individual variability in learning Chinese characters.

#### Multiple-choice Task

**Figure 2:**
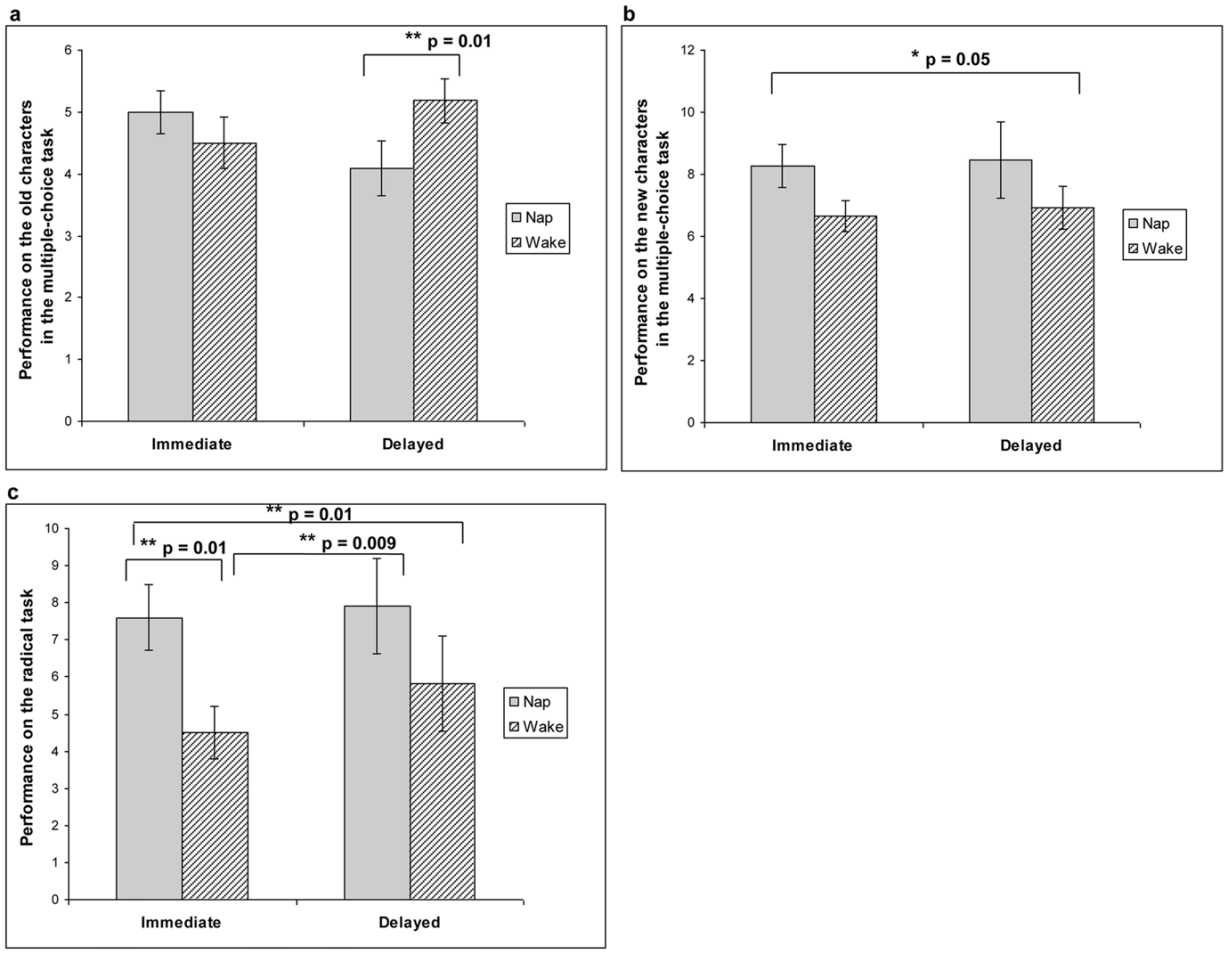
A summary of major findings. *(a)* Performance (i.e. number of correct answers) on matching old characters to their English meanings in the multiple-choice task. *(b)* Performance (i.e. number of correct answers) on matching new characters to their English meanings in the multiple-choice task. *(c)* Performance (scoring described in the Methods section) on the radical task. * Asterisks represent significant group difference and p-values are indicated.

Performance on the old characters and the new characters in this task was analyzed separately as they measured different kinds of memories – direct associative recognition memory and relational memory, respectively. Number of correct answers was used as a measure of memory in each case. A between-subject 2×2 ANCOVA revealed no significant main effects for either the nap condition (nap/wake) or the time condition (immediate/delayed) but a significant interaction (F_1,47_ = 5.50, p = 0.02, η^2^ = 0.11, *figure 2a*). There was no difference between the immediate nap (mean ± SEM: 5.00±0.35) and the immediate wake (4.50±0.42) group (F_1,27_ = 0.90, p = 0.35). On the other hand, there was a significant difference between the delayed nap (4.09±0.44) and the delayed wake (5.18±0.35) group (F_1,20_ = 7.63, p = 0.01). Particularly, the delayed wake participants recognized more English meanings for the old characters than the delayed nap participants.

A similar 2×2 ANCOVA was conducted for performance on the new characters. There was a marginally significant main effect for the nap condition (F_1,47_ = 3.99, p = 0.05, η^2^ = 0.08, *figure 2b*) such that the nap participants correctly matched English meanings to more Chinese characters they had not previously seen. Average scores for each group were as follows (mean number of correct responses ± SEM): immediate nap 8.27±0.68, immediate wake 6.64±0.50, delayed nap 8.45±1.23, delayed wake 6.91±0.69. There was no significant main effect for the time condition or interaction.

#### Radical Task

There was a significant main effect for the nap condition (between-subject 2×2 ANCOVA , F_1,47_ = 6.69, p = 0.01, η^2^ = 0.13, *figure 2c*). Specifically, the nap participants were better able to explicitly state the general concept of isolated radicals than their wake counterparts. Average scores for each group were as follows (mean score on the radical task ± SEM): immediate nap 7.60±0.88, immediate wake 4.50±0.71, delayed nap 7.91±1.28, delayed wake 5.82±1.29. There was no significant main effect for the time condition or interaction.

Examination of the nap effect within each time condition showed the immediate nap group (mean ± SEM: 7.60±0.88) having significantly better performance (p = 0.01) on the radical task than the immediate wake group (4.50±0.71). Although the performance of the delayed nap group (7.91±1.28) was numerically better than the delayed wake group (5.82±1.28), the difference did not reach significance. The variance was noticeably, although not significantly, greater in the delayed groups.

#### Time Effect

The nap groups and the wake groups were analyzed separately to further examine the time effect on performance. The delayed wake participants had marginally better recognition memory of the old characters than the immediate wake participants (mean ± SEM: 5.18±0.35 vs. 4.50±0.42.correct responses; F_1,23_ = 3.07, p = 0.09). There was no time effect on any other tasks in neither the nap nor wake participants.

#### Amount of External Interference

The possible explanation of nap effect by reducing external interference is addressed by comparing the delayed nap group to the immediate wake group because the two had the same amount of wakefulness between learning and testing. The delayed nap participants were superior in stating concepts represented by isolated radicals (delayed nap: 7.91±1.28 [mean ± SEM] vs. immediate wake group: 4.50±0.71; ANCOVA, F_1,23_ = 8.20, p = 0.009, η^2^ = 0.27). They also had more correct responses to the new characters but the difference did not reach significance (delayed nap: 8.45±1.23 vs. immediate wake group: 6.64±0.50; F_1,23_ = 2.22, p = 0.15). The lack of significance can be explained by the significantly greater amount of error variance in the delayed nap group (Levene's Test of Equality of Error Variances, p = 0.04). The two groups did not differ in recognizing the old characters (F_1,23_ = 0.48, p = 0.49).

## Discussion

The role of sleep in a flexible and adaptive form of memory called relational memory is one of the most important questions in this field of research. Using ideographical Chinese characters, the present study shows a beneficial effect of daytime napping on extracting a general concept from disparately learned but semantically related stimuli. While associative recognition memory of the learned Chinese characters was comparable when participants attempted to match the characters to their English meanings, the nap participants showed better relational memory as measured by the new characters. Moreover, the nap participants performed better when asked to explicitly state the concepts represented by isolated radicals. General learning ability, daytime sleepiness, intelligence, task difficulty or participants' motivation and interest in the tasks cannot explain the effect of napping on relational memory. The possible involvement of circadian factors is also eliminated because each nap group was accompanied by a wake control group that learned and was tested at the same time of the day. The results are consistent with the notion of sleep having a role in processes that integrate and reorganize memory traces.

Napping facilitates relational memory regardless of whether it occurs immediately after learning or after a delay. The findings corroborated the handful of studies consisting of this temporal element [13], [18]. Of equal importance, comparison between the delayed nap group and the immediate wake group showed the beneficial effect of daytime napping on relational memory, and is not a result of reduction in external sensory stimulation. The findings suggest an active, rather than passive, role of sleep in memory processing. The underlying mechanisms may involve neurophysiological, or neurochemical activities, or both. The activities may occur both on the system level such as global neural synchronization and desynchronization [19], temporally correlated hippocampal and cortical activities [20], and sleep-stage-dependent change in acetylcholine level [21], as well as on the cellular level such as neural reactivation [22], [23] and experience-dependent gene expression [24].

A striking difference between the present study and the majority of existing literature is the observation of the delayed nap group recognizing fewer old Chinese characters than the delayed wake group. It should be noted that there were only seven old characters used to assess associative recognition memory. This may render the analysis less meaningful. On the other hand, because the memory tasks were meant to be a surprise, participants were not explicitly instructed to avoid rehearsal of the learned materials. Unlike the majority of previous studies, participants were not allowed to leave the laboratory or engage in activities of their choice during the retention period. They watched non-arousing videos of ocean life while they were awake. It is possible that the delayed wake participants rehearsed the old characters during the retention period. They had the largest window for rehearsal, if that did occur. They did recognize the largest number of tested old characters out of the four groups although the difference is small and was not statistically significant when compared to the immediate nap and wake participants. With that said, it is equally possible that the processing of relational memory could interfere with the processing of the direct associative memory. A hallmark of the evolution of episodic memory is the retention of gist and eventual fading of specific details tied to the original experience. Sleep may accelerate this morphology of memory.

Our results make clear that sleep is important for the abstraction of generality. This is evident with even a brief period of daytime napping. Findings in the present study show the necessity of further research regarding the role of sleep in relational memory. It is important to identify the sleep-dependent mechanisms that facilitate relational memory to discern the effect of sleep on different relational memory tasks, and to delineate possible interaction between processing of relational memory and processing of memory that gives rise to relational links.
